# Effectiveness of pre-operative anaemia screening and increased Tranexamic acid dose policies on outcomes following unilateral primary, elective total hip or knee replacement: a statistical analysis plan for an interrupted time series and regression discontinuity study

**DOI:** 10.12688/f1000research.22962.2

**Published:** 2021-05-13

**Authors:** Ashley B. Scrimshire, Caroline Fairhurst, Catriona McDaid, David J. Torgerson

**Affiliations:** 1 Department of Health Sciences, University of York, York, UK; 2 Northumbria Healthcare NHS Trust, Wansbeck, UK

**Keywords:** Regression discontinuity, interrupted time series, quasi-experimental, anaemia, orthopaedics

## Abstract

Perioperative blood transfusion is associated with poorer postoperative outcomes following hip and knee replacement surgery. Evidence for the effectiveness of some measures aimed at reducing blood transfusions in this setting are limited and often rely on weak pre-post study designs. Quasi-experimental study designs such as interrupted time series (ITS) and regression discontinuity design (RDD) address many of the weaknesses of the pre-post study design. In addition, *a priori *publication of statistical analysis plans for such studies increases their transparency and likely validity, as readers are able to distinguish between pre-planned and exploratory analyses. As such, this article, written prospective of any analysis, provides the statistical analysis plan for an ITS and RDD study based on a data set of 20,772 primary elective hip and knee replacement patients in a single English NHS Trust. The primary aim is to evaluate the impact of a preoperative anaemia optimisation service on perioperative blood transfusion (within 7 days of surgery) using both ITS and RDD methods. A secondary aim is to evaluate the impact of a policy of increased tranexamic acid dose given at the time of surgery, using ITS methods.

## Introduction

Peri-operative red blood cell (RBC) transfusion is associated with poorer post-operative outcomes across surgical disciplines, including elective total hip (THR) and knee replacement (TKR) surgery ^1– 3^. Multi-modal patient blood management (PBM) programmes aim to reduce RBC transfusions and the associated complications. Two core elements of PBM are peri-operative tranexamic acid (TXA) and pre-operative anaemia screening and optimisation. However, debate exists around optimal TXA dose and there is a lack of high quality randomised controlled trials (RCT) into preoperative anaemia screening, with much of the evidence coming from pre-post design observational studies ^4^. The pre-post study design is common in the medical literature and causal associations are often inferred from them. However, they are subject to several flaws, including being unable to separate temporal changes from intervention effects and not accounting for regression to the mean ^5^. This frequently leads to over-estimation of a treatment effect and it has been described as the weakest observational study method ^5, 6^.

Although RCTs are considered the gold standard for evaluating changes in healthcare, they are not always feasible and the results may not always be generalisable to real world populations ^5, 7, 8^. A recent study comparing characteristics of patients recruited to peri-operative medicine RCTs with national registry data, observed significant differences in age, sex and ethnicity, potentially limiting the generalisability of RCT results ^9^. In addition, a RCT into preoperative anaemia optimisation may prove challenging as this practice is already recommended in multiple guidelines, as part of wider PBM programmes ^10– 12^. Where a RCT is not feasible quasi-experimental study designs such as interrupted time series (ITS) and regression discontinuity designs (RDD), can provide more robust evidence as they eliminate some of the threats to internal validity seen in pre-post studies.

The prospective publication of statistical analysis plans (SAP) for observational studies increases their transparency and likely their validity, as readers are able to distinguish between pre-planned and exploratory analyses ^13^. This paper, written prospective of any analysis being performed, provides the SAP for a quasi-experimental study using ITS and RDD methods on a large dataset of elective THR and TKR patients from a NHS Trust in England.

The primary aim of this study is to evaluate the clinical effectiveness of introducing a preoperative anaemia screening programme, which predominantly uses iron treatments, for patients undergoing primary, elective THR or TKR surgery. A secondary aim is to evaluate the clinical effectiveness of introducing a policy of increased intravenous TXA dose on induction of anaesthesia (15mg/kg [maximum 1.2g] increased to 30mg/kg [max. 2.5g]). Both interventions take place in the presence of a well-established, multi-modal, enhanced recovery programme, detailed elsewhere ^14^.

Although similar in design, ITS and RDD examine data from different perspectives. ITS is concerned with population-level changes over time, whilst RDD uses patient-level data to focus on effects on outcomes around intervention thresholds. These two analyses will provide complimentary results on the effectiveness of introducing a preoperative anaemia screening programme and an increased TXA dose of 30mg/kg in an NHS Trust ^15^.

## Statistical Analysis Plan

### Data source

Over time the orthopaedic department at Northumbria Healthcare NHS Foundation Trust (NHCT) has introduced a range of interventions aimed at improving post-operative outcomes for patients undergoing elective lower limb arthroplasty. These changes have been well documented in a series of published pre-post cohort studies ( Table 1) ^14, 16, 17^. This study will focus on the impact of two policy changes: 1) increased dose of TXA; and 2) introduction of preoperative anaemia screening. This study will not assess the impact of introducing an enhanced recovery programme (as the data are not available); however, this is included in Table 1 as it represents the time when the initial TXA policy (15mg/kg) was introduced (the pre-intervention period for the TXA analysis).

**Table 1.** Summary of previously published pre-post comparative cohort studies of interventions introduced in NHCT aimed at improving arthroplasty patient outcomes.

Author, year	Intervention	Pre-intervention	Post-intervention	Statistically significant outcomes reported by authors (p<0.05)
**Malviya, 2011** ^14^	Multimodal Enhanced Recovery Programme *	Jan 2004 – April 2008 (n=1500)	May 2008 – Nov 2009 (n=3000)	Reduced mortality, transfusions and LoS
**Morrison, 2017** ^16^	Increased dose of IV Tranexamic acid (30mg/kg max 2.5g)	May 2008 – July 2011 (n=2637)	Feb 2012 ** – Jan 2013 (n=1814)	Reduced transfusions
**Pujol-Nicholas,** **2017** ^17^	Introduction of pre-operative anaemia screening and optimisation pathway ***	Feb 2012 – Jan 2013 (n=1814)	Feb 2013 – May 2014 (n=1622)	Reduced transfusion, readmissions, critical care admissions, LoS and costs.

* Includes introduction of IV Tranexamic acid, 15mg/kg [max 1.2g], at induction of anaesthesia ** Policy introduced in August 2011. This study allowed a 6-month implementation period to ensure the change in practice had been adopted *** The impact of these policies are being assessed further in this study, with an updated dataset LoS = length of hospital stay

**Figure 1.**
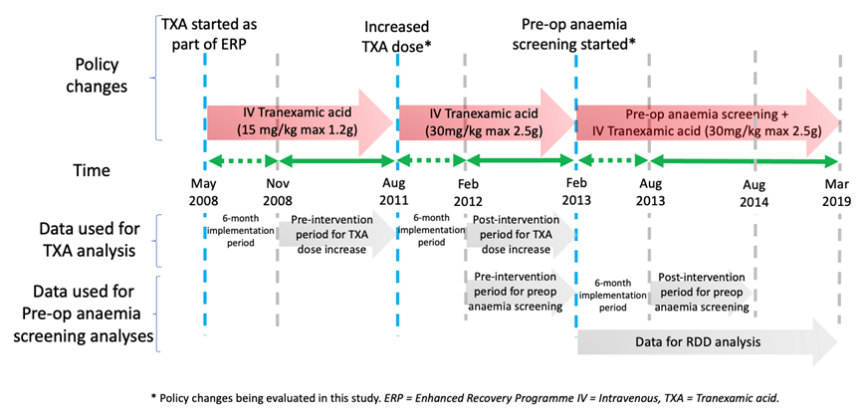
Timeline of patient blood management interventions introduced at Northumbria NHS Foundation Trust and data for analyses. ERP = Enhanced Recovery Programme IV = Intravenous, TXA = Tranexamic acid, ITS = interrupted time series, RDD = regression discontinuity design.

As part of on-going service evaluation, a large dataset of 20,772 primary, elective THR or TKR procedures performed at NHCT has been compiled. Procedures included in this dataset are those recorded with a procedure code (OPCS4) related to a primary hip or knee replacement, as detailed in Table 2. Procedures recorded with any other OPCS code are excluded from this dataset. The number of procedures included with each OPCS code will be reported with the results of this study.

**Table 2.** Procedure codes (OPCS-4) included in the dataset for this study.

OPCS code	Description
W371	Primary total prosthetic replacement of hip joint using cement
W381	Primary total prosthetic replacement of hip joint not using cement
W391	Primary total prosthetic replacement of hip joint NEC
W401	Primary total prosthetic replacement of knee joint using cement
W411	Primary total prosthetic replacement of knee joint not using cement
W421	Primary total prosthetic replacement of knee joint NEC

This includes data from hospital electronic record systems, such as the Patient Administration System and Blood Transfusion database, and a prospectively maintained database for the pre-operative anaemia screening service. The dataset includes patient demographics, comorbidities, pre-operative anaemia screening results (i.e. haemoglobin concentration, Hb), anaemia treatment given, operative details, post-operative complications, blood transfusions and length of hospital stay (LoS). The full dataset covers a time period from January 2008 to March 2019.

Ethical approval was not required as this is a retrospective study of routinely collected data. Local Caldicott guardian approval was given for use of these data. Data flow will be presented in a STROBE diagram in the resulting publication ^18^. Population characteristics (age, gender, comorbidities, type of surgery) and descriptive statistics will be presented in tables for the cohorts being studied. Analyses will be performed using R and RStudio (version R-3.6.2 for mac, R Core Team 2013, http://www.R-project.org/) on an intention to treat basis, and per protocol where possible. Results will be presented in terms of absolute and relative values with 95% confidence intervals where appropriate. Results will be considered statistically significant if the p-value ≤0.05.

### Outcomes

The primary outcome is the proportion of procedures requiring perioperative allogenic RBC transfusion (within 7 days of surgery). Secondary outcomes are the quantity of blood transfused per procedure (RBC units), LoS per procedure (in days), critical care admission rate per 100 procedure (within 30 days post-surgery) and emergency readmission rate per 100 procedures (within 30 days post-surgery) ^1, 2, 17^.

### Interrupted Time Series

ITS using segmented regression has several strengths over the pre-post study design. It controls for secular trends over time, provides powerful, easy to understand visual outputs, and may improve generalisability to the wider population ^7, 8, 19^. For this study, data are available to evaluate both policies described above in an ITS analysis.

The two interventions in this study were introduced at specific, well defined time points, allowing for clear separation of pre- and post-intervention periods. Figure 1 provides a timeline for the interventions in questions and Table 3 provides details of the planned analyses and time periods included in each. As shown in Figure 1, the same 12-month time period is used for both ITS analyses meaning they are not independent. However, there is insufficient time or data points to prevent this overlap, and this will be discussed as a potential limitation of the study.

**Table 3.** Planned ITS analyses.

Policy change	Dates	Analyses
Change in TXA dose policy	Pre-intervention 01/11/2008 – 31/07/2011 Excluded implementation (lag) period 01/08/2011 – 31/01/2012 Post-intervention * 01/02/2012 – 31/01/2013	PRIMARY – Population-level effects of TXA policy for all patients undergoing THR or TKR
Introduction of preoperative anaemia screening programme	Pre-intervention * 01/02/2012 – 31/01/2013 Excluded implementation (lag) period 01/02/2013 – 31/07/2013 Post-intervention 01/08/2013 – 31/07/2014 (sensitivity analysis 01/08/2013 – 28/02/2019)	PRIMARY – Population-level effects of anaemia screening programme for ***all patients*** undergoing THR or TKR (with equal number of data points pre- and post-intervention)
		SECONDARY - Effect on anaemic subpopulation ***eligible for any*** ***treatment*** as per anaemia pathway (Hb <120g/L, female or 130g/L, male) **, modelled against non-anaemic cohort
		SECONDARY - Effect on anaemic subpopulation ***eligible for iron*** treatment only (Hb 105–119g/L female, 115–129g/L male & ferritin 12- 100), modelled against non-anaemic cohort (excluding those with Hb <105 or <115 and those referred to GP/haematologist).
		SECONDARY - Effect on anaemic subpopulation ***given iron*** treatment only (as above but only those given iron), modelled against non-anaemic cohort (same exclusion as above) – per protocol analysis

* Post-intervention period for TXA policy is the same as the pre-intervention period for anaemia screening programme analysis. **includes GP referral, haematology referral or any form of iron

An early step in ITS analysis is to generate summary statistics for each time period and undertake simple pre-post comparisons ^20^. This will be performed in this study and later compared to the results from ITS and RDD analyses.

***Data description.*** ITS is said to work best with short-term outcomes that change quickly after implementation of an intervention or after a clearly defined lag period ^8^. This study is examining short-term outcomes; however, some delays to observed changes in outcomes after policy implementation are expected. The orthopaedic department has previously reported that a 6-month lag period was required to fully adopt the increased TXA dose policy ^16^. This same lag will therefore be incorporated into this ITS analysis. Regarding the introduction of the preoperative anaemia screening programme, staff running the anaemia service report that this started promptly on 01/02/2013, after detailed planning, and uptake was rapid. However, a lag to observed changes in outcomes will be inevitable due to surgical waiting list times. Comparing screening and surgery dates for the first 10 anaemic and 10 non-anaemic patients from the cohort shows all but one had their surgery within 6 months of screening. Therefore, a 6-month implementation (lag) period is also considered appropriate following introduction of the anaemia screening service ( Figure 1). Lag periods will be accounted for by excluding this data from the primary analysis ^21^. As individual procedure-level data are available, including data indicating whether or not anaemia screening was received, an alternative approach to handling the lag period for the analysis of the anaemia screening programme, could be to model the intervention as a continuous implementation variable ranging from 0 to 1, instead of as a binary (0/1) variable. This would allow the effect of the intervention to be modelled as a weighted average during the 6-month implementation period, which could be a more efficient use of the data available. A sensitivity analysis of this alternative method for handling the 6-month implementation period will be included in this study.

ITS requires sequential measures of the outcome, at regular intervals, before and after the intervention time points ^20, 21^. In keeping with many ITS studies, individual-level outcome data will be converted to, and presented as, proportions or means at monthly intervals and a segmented-regression analysis performed ^21^. ITS plots will be generated and visually inspected to determine if linear or non-linear regression modelling is appropriate. A minimum of 8 data points pre- and post-intervention are desirable ^20, 21^. It is expected the shortest time frame being analysed in this study will include 12 months/data points, thus surpassing this requirement. The power of an ITS analysis is increased if there are an equal number of data points pre- and post-intervention ^22^. In the case of preoperative anaemia screening there are expected to be 12 data points in the pre-intervention period but 60 in the post-intervention period. As such, to increase power of this analysis this period will be cut to include only 12 time points, after the 6 month lag period, for the primary analysis of the anaemia screening policy. Sensitivity analysis including all post-implementation data points will be conducted.

***Addressing threats to validity.*** Time varying confounders are the main threat to the validity of ITS studies ^21^. These are specific to each ITS study and are carefully considered later in this SAP. However, the most robust way to account for time varying confounders, even those that are unknown, is to model against a control group. This could either be a different population not exposed to the intervention or, if individual-level data are available, by splitting the data into two groups, one group targeted by an intervention and another that is not. In this study, data for a different group of patients is not available for either intervention. The TXA policy is targeted at all THR or TKR patients so this data cannot be split. However, the anaemia screening policy targets a specific subgroup of anaemic patients with treatments so the population can be split into two groups to increase the robustness of this analysis. As such the two interventions will be modelled separately: 1) the TXA intervention without a control group, and 2) the anaemia screening intervention with a control group.

As individual-level data are available, including data on the treatment received as part of the anaemia screening programme, there are several ways the ‘anaemic’ subgroup can be defined and analysed to give different information. These will be explored as secondary analyses. The primary analysis will include all patients undergoing THR/TKR in the time period, with no splitting into anaemic or non-anaemic subgroups, to assess the impact of introducing the anaemia screening programme on the entire population of patients undergoing THR or TKR. Previous pre-post studies, on an earlier version of this dataset, suggest the effect size is large enough for this to show through even though only a subpopulation of patients receive treatment for anaemia. Secondary analyses such as splitting the data into those defined as being anaemic (by the anaemia pathway being used) or non-anaemic, allow for modelling with a control group and evaluation of the effects of the screening programme on the subpopulation expected to receive some treatment, (i.e. iron, GP or haematology referral), and hence benefit from introduction of the anaemia pathway. It may also be possible to explore, specifically, the effects of iron treatment on the subpopulation of anaemic patients eligible (ITT) or actually given (PP) iron as part of the anaemia screening programme. Table 3 details the planned ITS analyses and, where appropriate, how the data will be split into two cohorts for secondary analyses. 

It is expected that the total number of procedures performed each month will be close to, or greater than, 100 for each time point in the primary analyses. However, as the data is split into cohorts (i.e. anaemic v non-anaemic), the number of procedures per month will reduce. This will likely increase the variability in the outcomes over time if reported as proportions (i.e. transfusion rate). Sensitivity analyses using event counts not rates will be conducted where the number of procedures per month drops below 30.

Time varying confounders specific to the primary outcome of this study may include other PBM interventions; those relevant will be discussed in turn. A restrictive blood transfusion policy was introduced Trust-wide in 2007 and has been unchanged since ^16, 17^. A multimodal enhanced recovery programme (ERP), including IV TXA on induction of anaesthesia, was introduced in May 2008. In keeping with other similar policy changes in this unit, a 6-month implementation period for the ERP is considered appropriate. To account for this, data from 1 ^st^ January 2008 to 31 ^st^ October 2008 will be excluded from this analysis. In addition, patient warming has been introduced locally ^23^ but a Cochrane review shows this does not affect surgical transfusion rates, so will not be considered any further ^24^. Intra-operative cell salvage has never been routinely used locally for the procedures being studied. To the best of our knowledge, no other relevant co-interventions have been introduced during the study period. Any unaccounted for, gradual changes in practice, would be detected in the pre-intervention slope of the TXA analysis and in the control group for the anaemia analysis.

Other considerations include changes in data coding, validity and reliability over time. The data for this study is considered reliable as it comes from a number of NHS Trust electronic databases detailed earlier in this paper. There have been no material changes to data collection methods or outcome reporting over the study period.

Changes in the population over time can also affect ITS reliability. There have been no known substantial changes in the population served by NHCT over the study period; however, to explore this within the data, tables of patient demographics (i.e. age, sex) for each of the pre- and post-intervention periods will be produced and examined for differences. Should substantial differences be identified, these characteristics will be incorporated into the final model as covariates, where possible.

This study includes a continually enrolled population, so is not subject to population attrition over time. Although no changes to diagnostic criteria for ischaemic heart disease (IHD) are known to have occurred during the study period, this comorbidity is specifically mentioned in the NHCT transfusion policy and lowers the threshold for considering transfusion. For completeness, rates of IHD will be plotted against time and visually inspected for any patterns, particularly around the time of the interventions. If required IHD will be included in the ITS modelling.

***Developing the model.*** Data will be inspected for outlier data points and, where identified, explanations will be sought and exclusion considered. First order lagged residuals will be included in the ITS models to account for autocorrelation ^20, 25^. The use of 6-month implementation periods in this study could be the same time between the lowest and highest points in an annual cycle, should seasonality affect outcomes. Although this is not expected in this instance, a sensitivity analysis including a lag of 12 time points (assuming seasonality) will be undertaken to test the robustness of the two primary analyses.

***Sensitivity analysis.*** An optimal model will be developed and described for these ITS analyses. The impact of decisions taken during this process such as inclusion/exclusion of outlying data points and autocorrelation adjustments will be tested in sensitivity analyses. Further analyses of data stratified by surgery type (THR or TKR) and/or by patient gender, will be conducted if data permits, as these may impact on outcomes.

### Regression discontinuity

RDD estimates the local average treatment effect when treatment decisions are based around a cut-off value for a continuous variable ^26^. For example, giving iron (the treatment) with the intention of reducing RBC transfusion and LoS (the outcomes) to patients whose Hb (the assignment variable) falls below a pre-defined cut-off of 120g/L for females or 130 g/L for males (the threshold). RDD makes use of this threshold and assumes that individuals who lie just above it belong to the same populations those who lie just below it, and assignment to treatment or not is considered random ^27^.

The main strength of RDD lies in its ability to achieve a balance of unobserved factors in patients that fall, by chance, either side of the threshold value, much like a RCT ^28^. The local nature of the effect examined in RDD can also be used in optimising threshold levels. In this case we may be able to examine if a threshold Hb of 120 or 130g/L may be more appropriate for females, as is being suggested in some studies ^12, 28– 30^. As the TXA policy affects all patients it is only possible to conduct a RDD analysis for the anaemia screening programme, using data since the inception of this programme (1 ^st^ February 2013, Figure 1).

**Figure 2.**
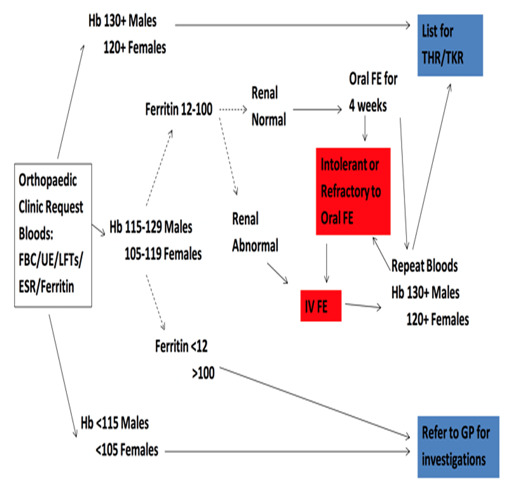
Northumbria Healthcare NHS Foundation Trust anaemia pathway demonstrating haemoglobin threshold values used to determine treatment ^17^.

***Data description.*** In this study the continuous assignment variable will be preoperative Hb concentration. The outcome assessment, for primary and secondary outcomes (listed above), are observed universally for patients who receive treatment or not. Details of how treatment is assigned has been previously reported, and is shown in Figure 2 ^17^. Notably the treatment thresholds are different for males (Hb 130g/L) and females (120g/L), so data will be stratified by gender for analysis. The treatment thresholds are based on World Health Organisation definitions for anaemia ^31^.

Treatment options from the pathway ( Figure 2) include iron (oral or IV) or referral for further investigation (to GP or haematologist) prior to surgery. This means it may be possible to examine the effects of the anaemia screening policy for all anaemic patients (including all treatments), but also to isolate the effects of treatment with iron by excluding patients who do not qualify for iron treatment (as per the pathway in Figure 2). Data permitting these variations will be explored. 

***Addressing threats to validity.*** Manipulation of treatment status by patients through manipulation of the assignment variable (Hb concentration) is highly unlikely. However, it is possible the reporting of the assignment variable could be manipulated by clinicians, although there is a protocol which healthcare professionals are required to, and report they, strictly adhere to. Nonetheless, steps will be taken to assess the internal validity of the data. A plot of assignment variable (Hb) against probability of receiving treatment will be created to inform if a sharp or fuzzy design is most appropriate ^29^. A histogram of the assignment variable (Hb) data will be visually inspected for bunching around the threshold values. This will also be tested using the McCrary density test ^32^. To test if groups either side of the threshold are comparable, summary statistics of non-outcome variables will be presented for those who fall just either side of the threshold. Formal testing (such as t-test for continuous variables) will be undertaken to assess for any statistically significant differences between groups either side of the threshold. It is predicated that some non-outcome variables such as age or comorbidities may affect outcome. As such sensitivity analyses incorporating these as covariates are planned regardless of the comparability of the two groups. If no differences between groups either side of the threshold are seen, this supports the assumption that assignment around the threshold is random and supports that there has been no manipulation of treatment status, similar to a RCT ^28^. If differences are seen, and it is possible the more anaemic patients (lower Hb) have more comorbidities and/or are older, then variables which are identified as being different between the two groups will be included as covariates in the final model. Similar to ITS, RDD is sensitive to co-interventions introduced around the threshold Hb value. There are no known co-interventions introduced locally for this patient cohort around the threshold Hb values.

***Developing the model.*** In graphical representations of the data, Hb will be divided into bins. Outcome data for this study is in the form of discrete variables (yes/no: transfused / readmitted / critical care admission; and number of inpatient days). As such, outcome data will be converted to a probability (i.e. risk of transfusion) or average (i.e. mean number of days) for each bin. To decide optimal bin size, plots of Hb and primary outcome (transfusion rate) will be generated for a range of bin sizes (i.e. 1, 2 or 5 g/L). Visual inspection of these plots will be used to rule out bin sizes that are clearly too wide or too narrow. For the remaining bin sizes, F-tests using *k*2 dummies and interactions will be performed to identify bin sizes that do not over smooth the data. From the remaining choices the widest bin size that is not rejected by either F-test will be chosen ^32^. The same bin size as chosen for the primary outcome will be used for plotting other outcomes to facilitate comparison. 95% confidence intervals will be plotted alongside the probabilities or averages where applicable.

Separate scatter plots of primary and secondary outcomes against Hb will be created. These will be inspected visually for a jump at the threshold value, indicative of a treatment effect. Data will be inspected for outlying data points and consideration given to exclusion. Sensitivity analysis with and without outlying data points will be performed.

As the sample size for this analysis is relatively small a parametric estimation of treatment effect will be used for the primary analysis using logistic regression ^32^. Length of stay will be considered as a continuous outcome. The F-test will be used to determine the optimal functional form of the parametric regression model. Starting with a simple linear model, a higher order term will be added to the model until the F-test is no longer statistically significant ^32^.

For the dichotomous outcomes of transfusion, readmission and readmission to critical care, logistic regression will be used. In all analyses, the simplest valid model will be preferred. Robustness checks of the models in which the outermost 1, 5 and 10% of data points are dropped will be conducted.

A nonparametric (local linear regression) approach will be taken in sensitivity analyses, within which the bandwidth will be determined by the cross-validation method ^32^. Further analyses with data stratified by surgery type (THR or TKR) will be conducted where data permits.

### Comparing ITS and RDD

Both ITS and RDD have significant advantages over the typical pre-post analysis often seen in medical literature. When an intervention is introduced rapidly and short-term outcomes are frequently assessed, ITS can be considered a sub-type of RDD in which the assignment variable is time and the cut-off occurs when the policy is introduced ^28^.

It is unusual to have a dataset amenable to both types of analysis; however, they provide different perspectives. Whilst both deigns share the strength of not being bound by the selective inclusion criteria of a RCT, thus potentially improving generalisability, they also have their limitations.

In the case of RDD, in order to ensure groups either side of the threshold are similar the focus is on an effect close to the threshold value. (i.e. female patients with Hb 119 or 121g/L are likely very similar, but those with Hb 90 or 140 are likely different in other, unmeasured parameters). This limits the generalisability of findings to values that lie far from the threshold. In the case of ITS the results can be impacted by several factors such as autocorrelation and unmeasured confounders, which we have attempted to address in the analysis design. Also, the findings from ITS can only indicate an associative not a causal relationship between intervention and outcomes, whereas RDD has the potential to demonstrate causation.

### Dissemination

Publication of study results will be sought in a high impact journal.

## Study status

Study data has been collected and analysis pending awaiting publication of this statistical analysis plan.

## Data availability

No data is associated with this article.
