# Supplementary material for: Effectiveness of pre-operative anaemia screening and increased Tranexamic acid dose policies on outcomes following unilateral primary, elective total hip or knee replacement: a statistical analysis plan for an interrupted time series and regression discontinuity study
Source: F1000Res. 2021 May 13;9:224. Originally published 2020 Apr 1. [Version 2] doi: 10.12688/f1000research.22962.2 (PMC7607480; doi:10.12688/f1000research.22962.2)
Supplement: Peer review history

# Peer review: Reviewer response for version 1

**DOI:** 10.5256/f1000research.25351.r72020

**Published:** 2020-11-02

**Authors:** Al Ozonoff

**Affiliations:** 1 Precision Vaccines Program, Division of Infectious Diseases, Boston Children's Hospital, Boston, MA, USA; 2 Department of Pediatrics, Harvard Medical School, Boston, MA, USA

The authors provide a prospective statistical analysis plan for a forthcoming study of orthopedic surgical cases from the UK National Health Service (NHS). The study uses quasi-experimental Interrupted Time Series (ITS) and Regression Discontinuity (RD) designs. The statistical analysis plan (SAP) is clear and well-written although there are some aspects that could be clarified. It is understood that many details of the model development will be determined upon examination of the data and thus a prospective statistical analysis plan should not be over-specified. However, there are some important elements of the modeling process that could be further explained in terms of what available methods might be considered.

**Specific comments follow:**

1. Data source. There could be more added to the section on data sources. The data set includes N=20,772 patients who have undergone primary elective THR or TKR surgeries at NHCT. Are there any surgeries excluded? A brief 1-2 sentences to state explicitly and formally the inclusion and exclusion criteria would be useful.
2. Interrupted Time Series. The exclusion of data from the six month period following each intervention seems overly conservative. Since the data are available for screening versus surgery time, the intervention can be modeled not as a binary (0/1) indicator but rather as a continuous implementation variable ranging from 0 to 1, estimated by the monthly proportion of surgeries for which patients received screening. Thus the effect of the intervention is modeled as a weighted average during the six month period following intervention which is a more efficient use of data and should provide a more precise estimate of the intervention effect.
3. The discussion of threats to ITS validity does not give much credit to the possibility that the patient population may change over the course of the study evaluation. While the authors note that there are no known changes in the overall population served by NHCT, it seems more plausible that there are shifts in demographics or other characteristics of the population receiving THR or TKR. Simple examinations of sex, age, and other clinical factors over the 11+ years of the study period seem warranted if only to verify that there are no major changes in the study population.
4. The discussion of threats to RDD validity could be sharpened. Most of the methods described involve visual inspection of graphical parameters with little formal testing planned. Comparability of groups on either side of the threshold might test formally the hypothesis of difference between groups as would be done for an RCT. It is not explained how bandwidth selection would address the threat to validity posed by incomparability, especially if an observed difference might be explained by manipulation of the assignment variable.
5. Describing model development, the bins for Hg will be chosen from options of 1, 2, or 5 g/L each with no explanation of what considerations are important nor how the data will drive the decision. Similarly, there should be detail provided on which ‘data driven methods’ will inform bandwidth selection.
6. There is no discussion of how to determine the functional form of the regression. What alternatives are considered if the relationship between Hg and outcome does not appear linear. There is a mention that non-linear models are considered without much insight into what methods are available in this case.

**Minor edits/corrections:**

1. Outcomes p3. Rates might be better specified with the appropriate denominator e.g. 30-day critical care readmission rate (per 1000 surgeries).
2. Data description p5. Typo ‘determin’ => ‘determine’.
3. Regression discontinuity p5. Typo ‘populationas’ => ‘population as’.
4. Addressing threats to validity p6. Typo ‘adhered to’ => ‘adhere to’.
5. Final phrase ‘Whereas RDD…’ is a fragment => combine with the previous sentence.

Is the study design appropriate for the research question?

Yes

Is the rationale for, and objectives of, the study clearly described?

Yes

Are sufficient details of the methods provided to allow replication by others?

Partly

Are the datasets clearly presented in a useable and accessible format?

Not applicable

Reviewer Expertise:

Statistics, epidemiology

I confirm that I have read this submission and believe that I have an appropriate level of expertise to confirm that it is of an acceptable scientific standard.

# Response

**Published:** 2021-03-26

**Authors:** Ashley Scrimshire

**Affiliations:** University of York, UK, UK

Thank you for taking the time to prove considered feedback on our article and for engaging in further discussion on your comments, this is very much appreciated. Your comments have been incorporated into the revised manuscript and a summary of our responses is given below.

**Comment: Data source. There could be more added to the section on data sources. The data set includes N=20,772 patients who have undergone primary elective THR or TKR surgeries at NHCT. Are there any surgeries excluded? A brief 1-2 sentences to state explicitly and formally the inclusion and exclusion criteria would be useful.**

****

**Response: **Thank you for your comment. This has been clarified in the text and Table 2 now presents the eligible procedure codes.

**Comment: Interrupted Time Series. The exclusion of data from the six month period following each intervention seems overly conservative. Since the data are available for screening versus surgery time, the intervention can be modeled not as a binary (0/1) indicator but rather as a continuous implementation variable ranging from 0 to 1, estimated by the monthly proportion of surgeries for which patients received screening. Thus the effect of the intervention is modeled as a weighted average during the six month period following intervention which is a more efficient use of data and should provide a more precise estimate of the intervention effect.**

****

**Response: **This is a very interesting point and thank you for engaging in further discussion on this. The paper has been updated. We now plan to include a sensitivity analysis modelling the intervention as a continuous implementation variable as suggested by the reviewer.

**Comment: The discussion of threats to ITS validity does not give much credit to the possibility that the patient population may change over the course of the study evaluation. While the authors note that there are no known changes in the overall population served by NHCT, it seems more plausible that there are shifts in demographics or other characteristics of the population receiving THR or TKR. Simple examinations of sex, age, and other clinical factors over the 11+ years of the study period seem warranted if only to verify that there are no major changes in the study population.**

****

**Response: **Thank you for highlighting this oversight, we agree with your comments. We will include comparison of key patient demographics and characteristics in the pre- and post-intervention groups. This has been included in the text.

**Comment: The discussion of threats to RDD validity could be sharpened. Most of the methods described involve visual inspection of graphical parameters with little formal testing planned. Comparability of groups on either side of the threshold might test formally the hypothesis of difference between groups as would be done for an RCT.**

****

**Response: **Thank you for your advice on these points. The manuscript has been updated to make this more robust. In particular we plan to generate tables and undertake statistical tests comparing non-outcome characteristics for groups either side of the threshold.

**Comment: It is not explained how bandwidth selection would address the threat to validity posed by incomparability, especially if an observed difference might be explained by manipulation of the assignment variable.**

****

**Response: **As detailed in the updated manuscript, manipulation of the assignment variable by patients is not considered likely in this scenario. However this will be explored within the data.

 We have now clarified our analysis plans in the manuscript. As such bandwidth selection is only relevant to our planned non-parametric sensitivity analysis, rather than the primary parametric analyses which will use all data. Sensitivity analyses in which the model incorporates predicted factors that may influence outcome such as age, comorbidities, will be undertaken. In addition, variables that are identified as being unbalanced between the two groups (i.e. as a result of possible manipulation of the assignment variable) will be included as covariates in further sensitivity analyses.

**Comment: Describing model development, the bins for Hg will be chosen from options of 1, 2, or 5 g/L each with no explanation of what considerations are important nor how the data will drive the decision. Similarly, there should be detail provided on which ‘data driven methods’ will inform bandwidth selection.**

**Response:** Agreed, we were not clear on our approach to this. The text has been updated. We intend to first plot data using a range of bin sizes and visually inspect these to rule out ones that are clearly too wide or too narrow. We will go on to conduct F-tests (using 2 *k *dummies and interactions) to identify bin widths that over smooth the data. From the remaining choices we will pick the widest bin size that is not rejected by either F-test. As for bandwidth selection, this is only relevant to our planned nonparametric sensitivity analysis. Here we intend to use the cross-validation method to inform bandwidth selection.

**Comment: There is no discussion of how to determine the functional form of the regression. What alternatives are considered if the relationship between Hg and outcome does not appear linear. There is a mention that non-linear models are considered without much insight into what methods are available in this case.**

****

**Response: **Agreed, we had not been clear on this, the text has now been updated. Our intentions are that after bin size has been selected plots will first be inspected visually. The F-Test approach will then be used to determine the functional form of the regression. Starting with a simple linear model and adding a higher order term until the F-test is no longer statistically significant. Robustness checks for this model in which the outer most 1,5 and 10% of data points are dropped will be conducted.

**Comment: Outcomes p3. Rates might be better specified with the appropriate denominator e.g. 30-day critical care readmission rate (per 1000 surgeries).**

****

**Response: **Agreed, this has now been updated in the text

**Comment: Typos/grammar:**

**Data description p5. Typo ‘determin’ => ‘determine’.**

**Regression discontinuity p5. Typo ‘populationas’ => ‘population as’.**

**Addressing threats to validity p6. Typo ‘adhered to’ => ‘adhere to’.**

**Final phrase ‘Whereas RDD…’ is a fragment => combine with the previous sentence**

****

**Response: **These have been corrected, thank you for highlighting.

# Peer review: Reviewer response for version 1

**DOI:** 10.5256/f1000research.25351.r70571

**Published:** 2020-10-08

**Authors:** David Reeves

**Affiliations:** 1 NIHR School for Primary Care Research, Centre for Primary Care and Health Services Research, Manchester Academic Health Science Centre (MAHSC), University of Manchester, Manchester, UK

It is very welcome to see a statistical analysis plan for an observational study submitted for publication, as this is relatively rare. It is also quite brave for the authors to do so: unlike an RCT, the form that the analysis of an observational dataset takes is largely dictated by the data available, and *a priori* plans that seem good in theory frequently need a major overhaul in the light of the actual data. Publishing a plan in advance risks making oneself a hostage to fortune if one’s good ideas subsequently turn out to be not so feasible in practice. However, what we do not know in the present instance, is how much of this plan is genuinely *a priori*, and how much based on data exploration and analysis already undertaken, though from the precise sample sizes and details presented I suspect quite a bit. Nonetheless, publication is still very worthwhile since the paper provides a level of detail probably not possible in a paper presenting the actual findings of the analysis, given the word-length restrictions of most publications.

 The authors present a mostly well-written and well-thought-out proposal that uses statistical methods of interrupted time-series and regression discontinuity analysis to evaluate the impact of changes in Hospital Trust policy around care for patients admitted for elective lower limb arthroplasty, on outcomes for those patients. The methods proposed are sound and it is good to see them being applied in this context. My comments below are largely concerned with improving the clarity around specifics of the analyses, issues around seasonal effects, and addressing autocorrelation.

 Table 1: Use of the terms “control cohort” and “intervention cohort” here is a little confusing, as the term “controls” is also used later under Addressing Threats to Validity, where it is applied to a sub-group of anaemic patients during the intervention period – i.e. a different control cohort. I would have preferred Table 1 to use terms such as “pre-intervention” and “post-intervention” to avoid confusion.

 If I understand Table 1 and Figure 1 correctly, there will be 3 ITS analyses, although the paper could be clearer about this in the text. Moreover, the majority of the intervention cohort for the first ITS (TXA started) will also be part of the control cohort for the second ITS (increased TXA) – since the date ranges overlap – and the intervention cohort for ITS 2 will be identical to the control group for ITS 3 (pre-op anaemia optimisation). If this is the case (or even if it is not) the authors need to clarify the situation here. Overlapping cohorts mean that the analyses will not be independent and may have implications for interpretation of the findings.

 For clarity I would like to have seen Figure 1 indicate the control cohort periods for each ITS, as well as the implementation and intervention periods. It took me some time work out how Table 1 related to Figure 1 in terms of the time-periods involved.

 Outcomes will be analysed in the form of monthly means or proportions. One issue here, which is not mentioned in the paper, is that the sample size will vary considerably over time. For the first ITS (TXA started) the control period covers approx 50 months and the sample size is 1500, implying a mean sample size of 30 patients per month – very small when the outcome is a proportion; whereas the intervention period is about 20 months with a total sample of 3000, indicating 150 per month. Thus outcome means/proportions will be far more variable over the control period. I haven’t checked, but the same may apply to the other ITS analyses. Ideally data-point variability should be taken into account in the analysis, and is something that the authors should at least mention and discuss the implications of, in the paper.

 Each ITS analysis will incorporate a 6-month implementation period between the pre and post periods, for which data will be dropped from the model. One concern here is the potential for an annual cycle in the data values. I cannot say if elective lower limb arthroplasty is subject to seasonal variation, but certainly hospital admissions for many other conditions are. The risk here is that 6 months can represent the time between the lowest and highest points in an annual cycle. Thus it is conceivable that at the end of the pre period, the cycle will be at it’s lowest point, but at the subsequent start of the post period, it will be at the top (or vice-versa). Particular care will need to be taken to evaluate whether any change in level or trend at this point can be explained by the presence of an annual cycle. The authors acknowledge the potential for seasonality in their discussion of autocorrelation (see below). However, I would like to see a specific sensitivity analysis designed to assess robustness against the threat of an annual cycle, regardless of the outcome of any tests for autocorrelation, given the use of a 6-month lag.

 Tests for autocorrelation, using the Durbin-Watson, are planned, using a lag of up to 12 time-points. However, these tests are likely to have very low power, given the numbers of data-points and the measurement error around the individual values (which at times will be very wide). To interpret a non-significant test as implying an absence of autocorrelation would be highly questionable. The data series will almost inevitably in reality possess autocorrelation, even if undetected by the DW, and in my view it would be better to conduct analysis under the assumption that autocorrelation is present. As I have suggested above, a sensitivity test against an annual cycle should be conducted regardless.

Is the study design appropriate for the research question?

Yes

Is the rationale for, and objectives of, the study clearly described?

Yes

Are sufficient details of the methods provided to allow replication by others?

Yes

Are the datasets clearly presented in a useable and accessible format?

Not applicable

Reviewer Expertise:

Statistics, Health research.

I confirm that I have read this submission and believe that I have an appropriate level of expertise to confirm that it is of an acceptable scientific standard.

# Response

**Published:** 2021-03-26

**Authors:** Ashley Scrimshire

**Affiliations:** University of York, UK, UK

Thank you for taking the time to provide considered and insightful feedback on our article. Your comments have been addressed in the revised manuscript. A summary of responses is given below.

**Comment: Table 1: Use of the terms “control cohort” and “intervention cohort” here is a little confusing, as the term “controls” is also used later under Addressing Threats to Validity, where it is applied to a sub-group of anaemic patients during the intervention period – i.e. a different control cohort. I would have preferred Table 1 to use terms such as “pre-intervention” and “post-intervention” to avoid confusion.**

****

**Response: **Agreed, this was unclear. Table 1 has now been updated and the terms “pre-intervention” and “post-intervention” have replaced “control cohort” and “intervention cohort” to avoid confusion.  This table also now outlines previously published, pre-post design cohort studies from this unit and does not outline the time periods for this analysis. A new Table 3 in the paper clearly outlines the time periods included in this analysis.

**Comment: If I understand Table 1 and Figure 1 correctly, there will be 3 ITS analyses, although the paper could be clearer about this in the text. Moreover, the majority of the intervention cohort for the first ITS (TXA started) will also be part of the control cohort for the second ITS (increased TXA) – since the date ranges overlap – and the intervention cohort for ITS 2 will be identical to the control group for ITS 3 (pre-op anaemia optimisation). If this is the case (or even if it is not) the authors need to clarify the situation here. Overlapping cohorts mean that the analyses will not be independent and may have implications for interpretation of the findings.**

****

**Response: **We agree that these figures and accompanying explanations could be clearer. The text has been updated to clarify that there will be two primary ITS analyses, plus secondary and sensitivity analyses. Figure 1 has been updated to clearly demarcate the pre- and post-intervention periods for each analysis. A new Table 3 also outlines the planned analyses and the time periods included in each.

**Comment: Outcomes will be analysed in the form of monthly means or proportions. One issue here, which is not mentioned in the paper, is that the sample size will vary considerably over time. For the first ITS (TXA started) the control period covers approx 50 months and the sample size is 1500, implying a mean sample size of 30 patients per month – very small when the outcome is a proportion; whereas the intervention period is about 20 months with a total sample of 3000, indicating 150 per month. Thus outcome means/proportions will be far more variable over the control period. I haven’t checked, but the same may apply to the other ITS analyses. Ideally data-point variability should be taken into account in the analysis, and is something that the authors should at least mention and discuss the implications of, in the paper.**

****

**Response: **Thank you for your comment. Data-point variability is expected, although not to the degree in the reviewer comment. Hopefully this is clearer now the time periods that are included in this study have been clarified in response to previous comments. Data-point variability has now been discussed in the amended text. The primary analysis will include all THR/TKR procedures in the dataset. Here the expected counts per month are 100 or more, so proportions will be used.

 For the secondary analyses, the data will be split into anaemic and non-anaemic sub-groups. Here, it is expected around 20-30% of patients per month will be anaemic, so the counts are expected to drop. In this instance analyses using proportions and counts will be undertaken.

**Comment: Each ITS analysis will incorporate a 6-month implementation period between the pre and post periods, for which data will be dropped from the model. One concern here is the potential for an annual cycle in the data values. I cannot say if elective lower limb arthroplasty is subject to seasonal variation, but certainly hospital admissions for many other conditions are. The risk here is that 6 months can represent the time between the lowest and highest points in an annual cycle. Thus it is conceivable that at the end of the pre period, the cycle will be at it’s lowest point, but at the subsequent start of the post period, it will be at the top (or vice-versa). Particular care will need to be taken to evaluate whether any change in level or trend at this point can be explained by the presence of an annual cycle. The authors acknowledge the potential for seasonality in their discussion of autocorrelation (see below). However, I would like to see a specific sensitivity analysis designed to assess robustness against the threat of an annual cycle, regardless of the outcome of any tests for autocorrelation, given the use of a 6-month lag.**

**Tests for autocorrelation, using the Durbin-Watson, are planned, using a lag of up to 12 time-points. However, these tests are likely to have very low power, given the numbers of data-points and the measurement error around the individual values (which at times will be very wide). To interpret a non-significant test as implying an absence of autocorrelation would be highly questionable. The data series will almost inevitably in reality possess autocorrelation, even if undetected by the DW, and in my view it would be better to conduct analysis under the assumption that autocorrelation is present. As I have suggested above, a sensitivity test against an annual cycle should be conducted regardless.**

****

**Response: **Thank you for your advice on this. The paper has been amended and the analyses will assume autocorrelation is present and a sensitivity analysis assuming seasonality has also been incorporated.
